# Comparison of antimicrobial efficacy and therapeutic index properties for common wound cleansing solutions, focusing on solutions containing PHMB

**DOI:** 10.3205/dgkh000528

**Published:** 2024-12-16

**Authors:** Fergus Watson, Rui Chen, Jeanne Saint Bezard, Steven Percival

**Affiliations:** 1 5D Health Protection Group Ltd, Liverpool, UK

**Keywords:** therapeutic Index, antibiofilm efficacy, wound/surgical irrigation, cytotoxicity, polyhexanide, PHMB, poloxamer, ESKAPE pathogens, surgical side infection, SSI

## Abstract

**Background:**

Surgical site infections (SSIs) have been shown to increase patient morbidity and mortality, impact on quality of life and place a significant economic burden on healthcare systems worldwide. Irrigation using wound cleansing and antiseptic effective solutions during surgical procedures is a key part of SSI prevention. The optimal solution would have minimal cytotoxicity to the patient while maintaining a minimum concentration required for antimicrobial activity necessary to prevent opportunistic pathogens and biofilm formation.

**Method:**

A variety of common wound irrigation products, including polyhexanide in various concentrations and compositions, iodine and hypochlorous acid-based solutions, were tested and compared for their activity against pathogens according to the ESKAPE group of nosocomial relevant microbes. The antimicrobial efficacy of the solutions was tested against planktonic cells using a time-kill assay. Its minimal bactericidal concentration (MBC) and its cytotoxicity against mouse fibroblast cells were determined. Finally, the Therapeutic Index (TI) was compared and the biofilm activity of a selected solution containing 0.1% polyhexanide (PHMB) was tested.

**Results:**

Irrigation solutions containing 0.1% PHMB demonstrated rapid inactivation against planktonic cultures, achieving >4 lg reduction within 60 seconds. When comparing the TI of all irrigation solutions tested, the combination of 0.1% PHMB and poloxamer as an additive showed the best results in killing nosocomial pathogens and also to be less cytotoxic to mammalian fibroblasts, as demonstrated for PREVENTIA^®^ Surgical Irrigation. When exposed to five single-species biofilms, PREVENTIA^®^ Surgical Irrigation showed a 3 lg reduction (average) after 60 minutes; this was supported by microscopy showing significant biofilm disruption and an abundance of non-viable microcolony formations.

**Conclusion:**

This study highlights the impact of irrigation solutions containing PHMB. It also demonstrated the effect of using different concentrations of PHMB in combination with surfactants as additives. The combination of 0.1% PHMB and poloxamer as a surfactant demonstrated effective benefits in eradicating established biofilm combined with a relatively high Therapeutic Index (TI), indicating low cytotoxicity and high bactericidal activity.

## Zusammenfassung

**Hintergrund:**

Surgical Site Infections (SSI) erhöhen die Morbidität und Mortalität der Patienten, beeinträchtigen die Lebensqualität und stellen für die Gesundheitssysteme weltweit eine erhebliche wirtschaftliche Belastung dar. Die Spülung mit antiseptisch wirksamen Wundspüllösungen zur Säuberung während chirurgischer Eingriffe ist ein wichtiger Bestandteil der SSI-Prävention. Die optimale Wundspüllösung sollte eine möglichst minimale Zytotoxizität für den Patienten aufweisen und gleichzeitig in so minimaler Konzentration wie möglich eine antimikrobielle Wirkung aufweisen, die zur Verhinderung von Infektionen mit opportunistischen Erregern und der Bildung von Biofilm erforderlich ist.

**Methode:**

Eine Reihe gängiger Wundspüllösungen, darunter Lösungen auf Basis von Polihexanid (PHMB) in verschiedenen Konzentrationen und Zusammensetzungen sowie Lösungen auf Iod- und Hypochloritsäurebasis, wurden hinsichtlich ihrer Aktivität gegen Erreger der ESKAPE-Gruppe nosokomial relevanter Mikroben getestet und verglichen. Die antimikrobielle Wirksamkeit der Lösungen wurde in einem Time-Kill-Assay gegen planktonische Zellen getestet. Die minimale bakterizide Konzentration (MBC) und die Zytotoxizität gegen Maus-Fibroblasten wurden bestimmt. Schließlich wurde der therapeutische Index (TI) der Wundspüllösungen verglichen und die Aktivität gegen Biofilm einer ausgewählten Lösung mit 0,1% PHMB getestet.

**Ergebnisse:**

Spüllösungen, die 0,1 % PHMB enthielten, zeigten eine schnelle Inaktivierung von planktonischen mikrobiellen Zellen mit einer Reduktion um >4 lg-Stufen innerhalb von 60 s. Beim Vergleich der TI aller getesteten Spüllösungen zeigte die Kombination aus 0,1 % PHMB und Poloxamer als Additiv die besten Ergebnisse bei der Abtötung nosokomialer Krankheitserreger bei gleichzeitig geringer Zytotoxizität gegenüber den Säugetierfibroblasten. Bei der Exposition gegenüber fünf speziesreinen Biofilmen zeigte PREVENTIA^®^ Surgical Irrigation nach 60 min eine Reduktion um durchschnittlich 3 lg-Stufen, was mikroskopisch bestätigt wurde. Es zeigten sich eine signifikante Degeneration des Biofilms sowie nicht-vitale Mikrokolonien im Vergleich zu einem unbehandelten Biofilm.

**Schlussfolgerung:**

Die Studie unterstreicht die Wirksamkeit von chirurgischen Wundspüllösungen auf Basis von PHMB. Darüber hinaus wurde der Effekt der Verwendung unterschiedlicher Konzentrationen von PHMB in Kombination mit Tensiden als Zusatzstoffe gezeigt. Die Kombination aus 0,1 % PHMB und Poloxamer als Tensid erwies sich als wirksam bei der Beseitigung eines etablierten Biofilms und wies einen relativ hohen therapeutischen Index (TI) auf, was auf eine geringe Zytotoxizität und eine hohe bakterizide Aktivität hinweist.

## Introduction

Disruption of the skin can result in unwanted opportunistic pathogenic microorganisms from entering the body and forming a recalcitrant biofilm and so increase a wound´s susceptibility to inflammation and infection. Surgical procedures are key examples of how these opportunistic pathogens such as *Staphylococcus (S.) aureus*, *Pseudomonas aeruginosa* and *S. epidermidis*, often part of the patient’s own microbiome, may enter the body and result in a surgical site infection (SSI) [1]. The United States Centre for Disease Control and Prevention (CDC) classifies an SSI as a superficial incisional infection, which involves only skin and subcutaneous tissue; a deep incisional infection; which involves deeper soft tissues of the incision; and an organ or space infection, which involves any part of the body which was opened or manipulated during a surgical procedure. SSI, a wound infection that has occurred within 30 days of a surgery, can occur in 11% of general surgical patients [2], [3]. Once established, the treatment of SSIs can be a challenge and often requires a multi-faceted approach; this places a burden on the patients and their care, resulting in high therapeutic costs, reduced quality of life and increased morbidity [4]. 

Biofilm formation plays a vital role in the pathogenesis of SSIs. The attachment of microorganisms, such as bacteria or yeasts, to the surrounding tissue, hardware and wound dressing is considered as the first stage of infection [5]. Once attached, microbial cells begin to proliferate and aggregate on the wound surface or deep within the wound to form multilayer three-dimensional structures referred to as microcolonies or early stage biofilm [6]. Microorganisms organised within biofilms enter a state of reduced metabolic activity. In some instances, this can result in the dispersal of free floating or planktonic microorganisms into the host tissues triggering an immune response causing inflammation, patient discomfort and loosening of any implant through osteoblast apoptosis [7], [8]. Whilst there are many strategies for treating SSI including further surgical intervention such, as debridement or replacement of mobile exchangeable parts, or antimicrobial therapies, such as targeted or broad spectrum antibiotic usage, prevention in the first instance is critical [9]. Prevention strategies can often centre upon the use of antiseptic solutions used to cleanse the wound environment during surgical procedures. These solutions are routinely used on clean wounds that have been debrided as a prophylactic intraoperative incisional wound irrigant [10], [11].

To avoid SSI from occurring guidance from the CDC and the World Health Organisation (WHO) have recommended the use of intraoperative wound irrigations with povidone iodine solution [12], [13], [14]. Since then, irrigation solutions have gained popularity due to their ability to inactivate microbes and disrupt biofilms within the wound. Today a variety of products are available [15], [16]. These solutions differ in relations to their antimicrobial active ingredient, other components/additives and functions. Whilst irrigation of infected wounds is not a new topic there is limited data on the efficacy of SSI prevention strategies using irrigation solutions containing antibacterial agents [10], [17]. Most surgical irrigation solutions are classified as medical devices and are subject to regulations that limit the use of additives such as bactericides. It is also a challenge for manufacturers to find the right balance between medical device and pharmaceutical, with pharmaceuticals being subject to significantly higher regulatory requirements and approval processes [10]. In 2010, the following ranking for the treatment of chronic wounds was derived based on the results of a comparative testing according to DIN 58940-7, DIN 58940-8, DIN EN 1040 and 1275: PHMB >octenidine >chlorhexidine >triclosan >PVP-iodine [18]. In 2018, a wound antisepsis consensus revealed that polyhexanide (polyhexamethylene biguanide, PHMB) was the most common agent chosen by surgeons for infected wounds. PHMB is a relatively new antiseptic candidate for use in surgical irrigation solutions, as it has been used across multiple industries from food and cosmetics since the last five decades. PHMB has broad antimicrobial effects with bactericidal activity against both Gram-positive and Gram-negative bacteria, as well as yeasticidal activity making it an ideal agent against bacteria and fungi in clinical use [19], [10].

In this study, we determined the efficacy of some commonly used wound irrigation solutions towards a variety of microbes growing within both the planktonic and biofilm phenotypic states, in conjunction with cytotoxicity analysis to determine the therapeutic index (TI) value. All used bacteria strains are known to cause nosocomial/healthcare-associated infections and be part of the so called ESKAPE pathogens that develop multidrug resistance and virulence. The study focuses on the use of PHMB irrigation solutions and their ability to both inactivate planktonic microorganisms and prevent biofilm formation, as PHMB is described as a broad-spectrum antimicrobial agent with no known microbial resistance. 

## Method

### Test samples

Different wound irrigating solutions, currently available on the market, were tested in this study, 0.9% saline solution and phosphate buffered saline (PBS) as controls. The wound irrigating solutions included PREVENTIA^®^ Surgical irrigation, referred to here as PHMB-Poloxamer (water, poloxamer and polyhexanide; 0.1%), Prontosan™, referred to here as PHMB-Betaine (purified water, betaine surfactant, polyhexanide; 0.1%), Lavasorb™, referred to here as PHMB-Macrogolum (purified water, polyhexanide; 0.04%, sodium chloride, potassium chloride, calcium chloride dihydrate, macrogolum 4000), Betaisodona™, referred to here as PVP-Iodine (povidone, hydrogen iodide, and elemental iodine), and Granudacyn™, referred to here as HClO/NaClO (sodium hypochlorite, hypochlorous acid).

### Test organisms 

The efficacy of wound irrigation solutions was tested against single species models consisting of bacterial strains, *Pseudomonas (P.) aeruginosa* ATCC 15442, *Staphylococcus (S.) aureus* ATCC 6538, methicillin-resistant *S. aureus* (MRSA) ATCC BAA-43, *Acinetobacter (A.) baumannii *clinical isolate No. 154846, and *Candida (C.) albicans* ATCC 10321. Bacterial strains were set up by inoculating tryptone soy agar (TSA) (Oxoid, UK) with working cultures stored at –80°C and incubated for 24 hours at 37°C in a static incubator. *C. albicans* was cultured on Sabouraud dextrose agar (SDA) (Oxoid, UK) for 48 hours at 30°C. Following incubation time, strains were adjusted to ~1×10^8^ colony forming units (cfu)/mL using a spectrophotometer (Jenway model 7205) standard.

### Materials 

Reagents and media were prepared according to the manufacturer’s instructions. Dey-Engley Neutralizing broth, neutralizing components include Sodium thioglycolate, sodium thiosulphate, sodium bisulphite, soya lecithin and polysorbate 80, (Millipore, UK) was used to neutralise all the wound irrigating solutions throughout the study. Tryptone Soy Agar (TSA) (EO Labs, UK) and Sabouraud dextrose agar (SDA) (EO Labs, UK) were used as culture medium. Tryptone soy broth (TSB) (Oxoid, UK) and Sabouraud dextrose broth (SDB) were prepared for inoculation of the models; and phosphate buffered saline (PBS) (Oxoid, UK) was used for serial dilution of the samples.

### Time kill study 

After recultivation, all the bacteria and the yeast suspensions were then further diluted 1:100 in PBS to form an adjusted culture of ~1×10^6^ cfu/mL. Using an Eppendorf, 100 µL of each microbial suspension was then exposed to 1 mL of each antimicrobial treatment. After which, a 100 µL of each test suspension was taken at 1 minute, 3 minute, 5 minute, 10 minute, 15 minute, 30 minute and 24 hour time intervals, in triplicate, and added to 0.9 mL of neutralising broth. These were then vortexed for 10 –30 seconds before being plated onto TSA and SDA. TSA plates were incubated overnight at 37°C for the bacterial strains, and SDA plates were incubated at 30°C for 48 hours for the fungal strains. Following incubation, counts were enumerated for each of the time points to quantify viability, cfu/mL.

### Minimum bactericidal concentration (MBC) 

To determine the minimum bactericidal concentration (%) of each irrigation solution, the products were diluted 1:2 with PBS before being serially diluted in 96-well plates. To prepare serial dilutions of the test products, 100 µL of each product was added to column 1 of the 96-well plate, 50 µL of PBS was added to column 2–11 and 100 µL was added to column 12 as a sterility control. A 2-fold serial dilution was then performed in columns 1–10 by transferring 50 µL from column 1 into column 2 and mixing, then 50 µL from column 2 into column 3 and so on.

All the bacterial and yeast suspensions were then diluted in PBS to form an adjusted culture of ~1×10^6^ CFU/mL. Then, 50 µL of the adjusted microbial suspension was added to columns 1–11, with column 11 being a positive control. Plates were wrapped in parafilm to avoid evaporation of the product and incubated at 37°C for 60 minutes.

Following incubation, each column was diluted 1:2 in neutralizing broth, and 100 µL samples of each well was plated onto TSA for the bacterial strains, and SDA for the yeast strain, and left to dry for approximately 30 minutes. Once dry, TSA plates were incubated at 37°C overnight and SDA plates were incubated at 30°C for 48 hours. Following incubation, the plates were examined visually for growth and the MBC was denoted as the lowest concentration of the treatment solution in which there is a complete absence of colony growth on the agar plate.

### In vitro cytotoxicity against L929 cells 

This protocol was conducted in accordance with an adaptation of the British Standard European Norm ISO 10993 for the biological evaluation of medical devices. L929 cells, a mouse fibroblast cell line, was maintained in Dulbecco’s modified Eagle’s medium (DMEM) GlutaMAX^TM^ (Gibco^TM^, ThermoFisher Scientific, UK) supplemented with 10% fetal bovine serum (FBS) and 100 units/mL penicillin-streptomycin. Cells were cultured at 37°C and 5% C0_2_ until approximately 90% confluence before passage. After incubation, the cells were then seeded at 1.5×10^4^ cells per well in 96-well plates and incubated at 37°C for 18 hours. After incubation time, the growth medium was removed out of the 96-well plates, and 30 µL fresh medium was added to each well. Each irrigation solution was serial diluted with PBS to make varying concentration solutions, before adding 170 µL of each dilution to individual wells containing the cells, bringing the total volume to 200 µL per well. After 60 minutes exposure at 37°C, the solutions were aspirated and 100 µL of fresh growth media and incubated for 24 hours at 37°C.

Following incubation, 20 µL of the MTS-PMS assay solution, which was prepared immediately before use, was dispensed in each well and incubated at 37°C for 120–135 minutes; after which the optical density (OD) was measured at 490 nm. A reference wavelength at 680 nm was also measured to eliminate background signal caused by cell debris and other nonspecific absorbance.

The percentage of cell viability was calculated by:


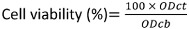


ODct=the mean value of the measured optical density (OD) of test article at 490 nm subtracted from the OD of test article at 680nm

ODcb=the mean value of the measured OD of the control blank subtracted from the OD of test article at 68 nm

### Relative therapeutic index 

The relative therapeutic index of a tested irrigation solution is defined as the ratio of the concentration required to achieve 50% cell toxicity (CT50) divided by the MBC.

### Antibiofilm assay 

All the bacteria suspensions were further diluted with 3 mL TSB to form an adjusted culture of ~1×10^3^ cfu/mL. For *C. albicans*, an adjusted culture of ~1×10^6^ cfu/mL was used.

Using the adjusted cultures, individual 96-well plates were inoculated with 150 µL of each strain. A peg lid was then placed on top of the plate and sealed with parafilm to prevent evaporation. The plates were then incubated at 37°C for 18 hours at 120 rpm. For *C. albicans*, individual 96-well plates were inoculated with 150 µL before a standard lid was on top of the plate and sealed with parafilm to prevent evaporation. The plates were incubated at 30°C for 48 hours at 120 rpm. 

Following incubation, each well was washed three times with PBS and then 200 µL of the irrigation solutions previously diluted at 1:10 with PBS were transferred to the wells for an exposure time of 60 minutes. Afterwards, the test products were removed from the 96-well plates, and 200 µL of the neutralising broth was added to the plates to quench the antimicrobial activity of the test solutions. 

Once quenched the plates were sonicated for 30 minutes at full power before being serially diluted 1:10 with PBS and plated out onto TSA, for bacterial cultures, and SDA, for fungal cultures before being incubated at 37°C and 30°C, respectively, for up to 48 hours. Following incubation, the plates were enumerated to determine viability of the remaining biofilm, cfu per surface area (cm^2^).

### Statistical analysis 

Raw data counts were inputted into Microsoft Excel and lg cfu/mL calculated. To determine if there was a statistical difference between the untreated controls and treated samples a t-test was conducted.

The total number of surviving cells were compared with suitable controls to determine the lg reductions achieved. The lg reduction was calculated using this formula:

lg reduction=control lg cfu/cm^2^–treatment lg cfu/cm^2^

### Microscopic analysis

A culture of *P. aeruginosa* ATCC 15442 was set up by inoculating 10 mL of TSB with a single colony and incubating at 37°C and 125 rpm in an orbital shaking incubator overnight, after which the culture was adjusted to ~1×10^6^ cfu/mL. LabTek 4-well chamber slides were inoculated with 1 mL of the adjusted culture per chamber and incubated for 24 hours at 37°C and 125 rpm in an orbital shaker.

After incubation, the medium was removed and the biofilm gently washed with PBS to remove any planktonic bacteria. Then 0.5 mL of PHMB-poloxamer irrigation solution was added directly on top of the biofilm and incubated at 37°C for 60 minutes. Next the irrigation solution was removed and the biofilm was washed with PBS to remove all remaining irrigation solution before being stained with the LIVE/DEAD™ Baclight™ fluorescent stains adjusted to a final concentration of 2.5 µM of SYTO 9^®^ (green fluorescence, live cells) and 27.5 µM of propidium iodide (red fluorescence, dead cells). The samples were incubated at room temperature in the dark for 15 minutes, before the stain was then aspirated, and the biofilms were gently washed with PBS. Following the wash step, 20 µL of PBS was added to each well to ensure the biofilm did not dehydrate during imaging. The stained biofilms were visualised with an LSM 780 Zeiss confocal laser-scanning microscope (CLSM) with a 60x oil objective. All images were taken under identical conditions. The acquired images and the subsequent production of the 2.5D profiles were carried out with Fiji – ImageJ software.

## Results

### Time kill assay 

**Figure 1:**
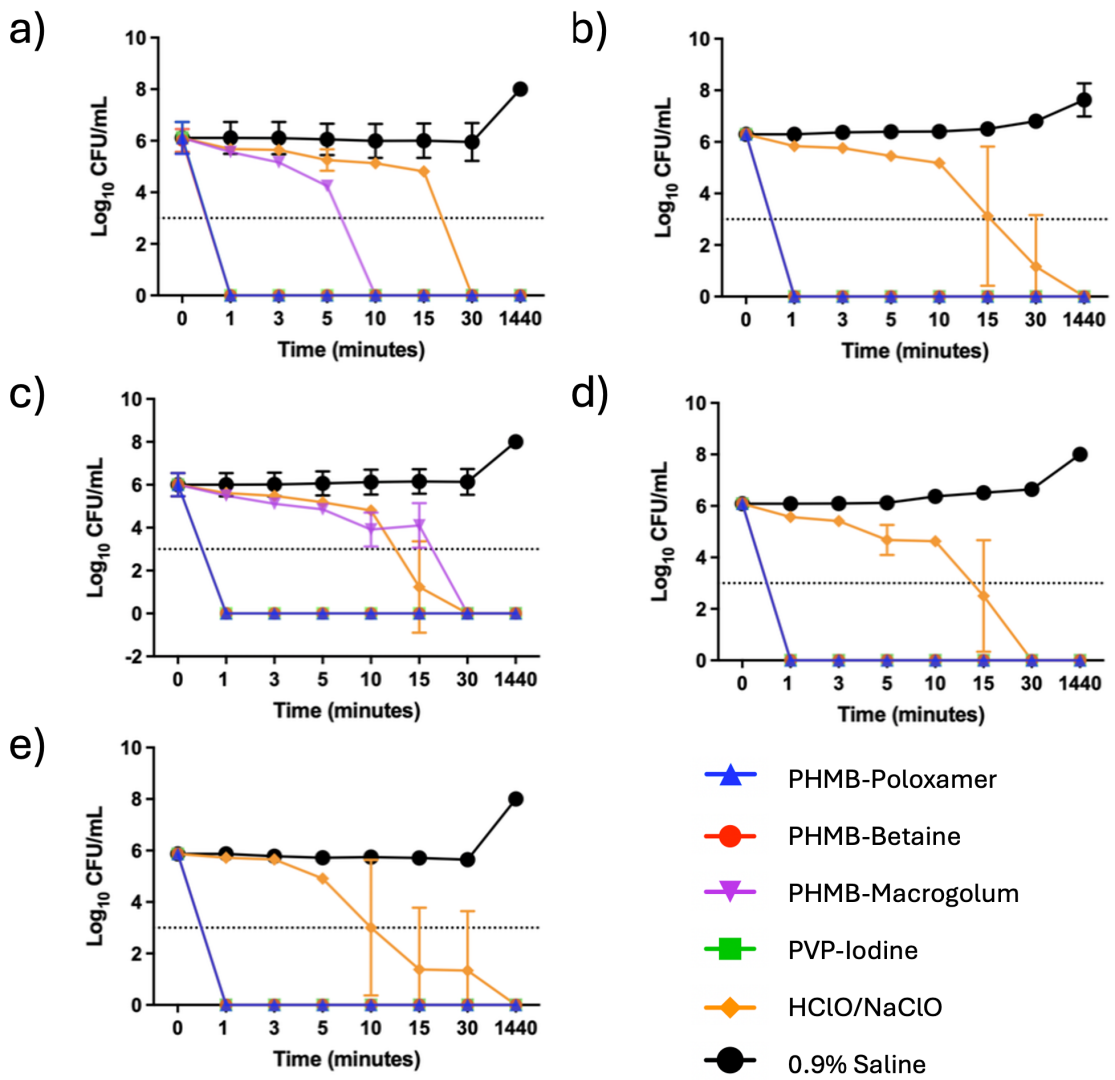
The time kill curves (n=3) for the single species planktonic models using a) *P. aeruginosa*, b) MRSA , c) *S. aureus*, d) *C. albicans* and e) *A. baumanni* (the limit of detection is indicated by the dotted horizontal line)

Upon exposure to the irrigation solutions all planktonic microorganisms, P*. aeruginosa, S. aureus*, MRSA, *C. albicans* and *A. baumannii*, demonstrated no viable growth after 24 hours indicating complete inactivation of the cultures (Figure 1 (Fig. 1)). All the irrigation solutions tested showed a lg 3 reduction of ESKAPE microbes, but there were dramatic differences in the time required. PHMB-poloxamer, PHMB-betaine and PVP-iodine all showed strong antimicrobial properties, which even caused a reduction of more than 3 lg in all species within 1 minute. In comparison HClO/NaClO required between 30 minutes and 24 hours to achieve equivalent levels of inactivation. PHMB-macrogolum also demonstrated a strong potential to reduce used microbes, similar to the other PHMB-containing products, except against *S. aureus* strains in this study.

### Therapeutic index 

The therapeutic index (TI) describes the ratio between the concentration required for efficacy on a microorganism and the toxicity of the same substance on the mammalian cells (Table 1 (Tab. 1) and Table 2 (Tab. 2)). A low TI indicates a weak antimicrobial activity and/or strong cytotoxicity, while a high TI indices for the irrigation solutions shown here were assessed using mouse fibroblast cells (L929). Upon exposure, on average, povidone-iodine exhibited the lowest TI value (average, 0.95±0.00). In comparison the highest TI value was recorded for HClO/NaClO (average, 10.54±5.26) (Table 3 (Tab. 3)).

### Antibiofilm efficacy 

**Figure 2:**
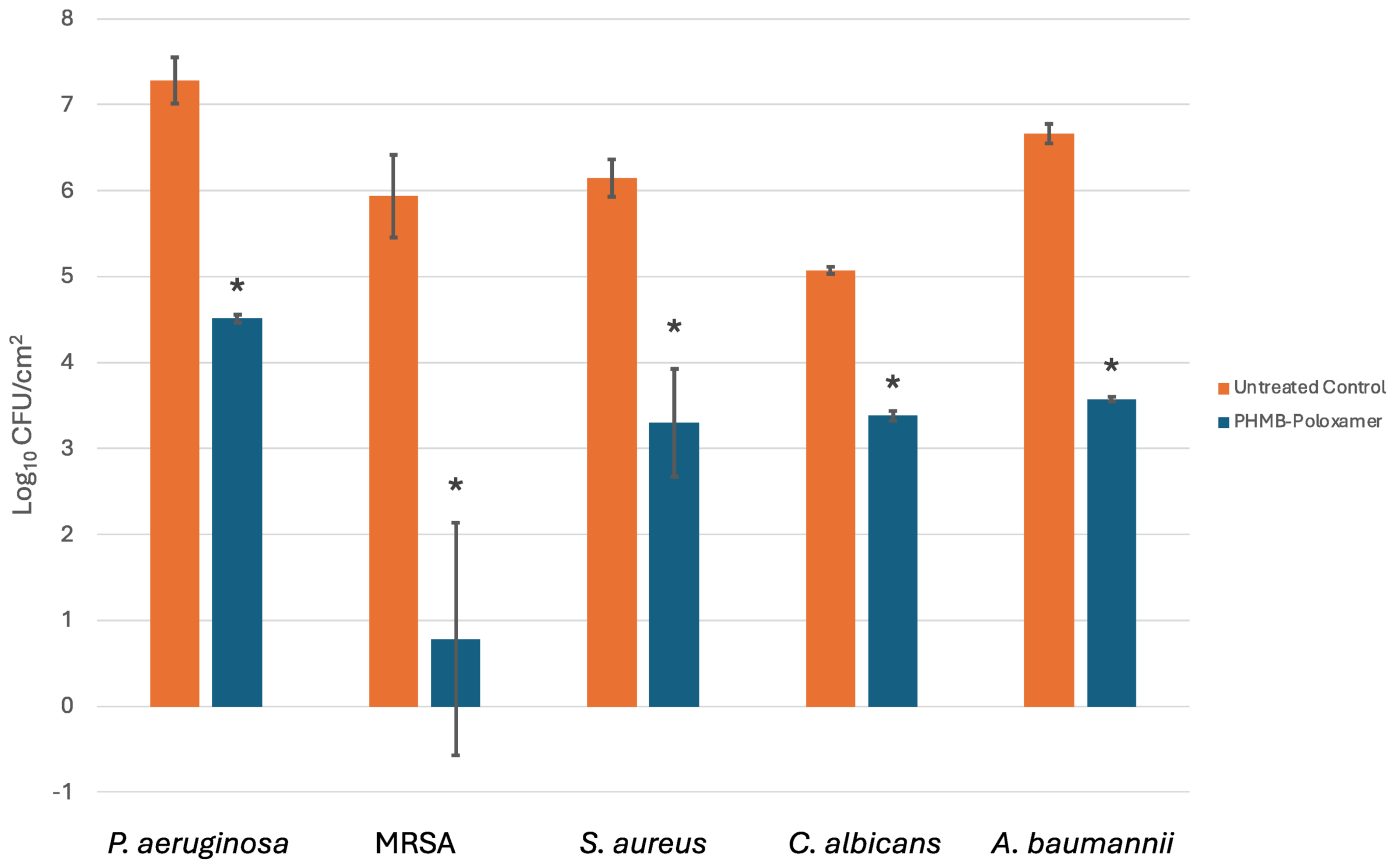
A comparison of the microbial concentration levels (lg cfu/cm^2^) for untreated and treated biofilms after 60 minutes exposure to PHMB-poloxamer (n=3). A significant reduction was detected for all treated biofilms in comparison to the untreated controls (*: p<0.05).

Due to its enhanced therapeutic indices, the antibiofilm efficacy of PHMB-poloxamer irrigation was investigated against biofilms formed using microtiter plate assays. Across all microorganisms tested the biofilm density (lg cfu/cm^2^) was, on average, 6.22±0.80 and an average reduction of 3.11±1.40 was exhibited after 60 minutes exposure with a statistically significant drop across all data sets (p<0.05) (Figure 2 (Fig. 2)).

### Microscopic analysis 

**Figure 3:**
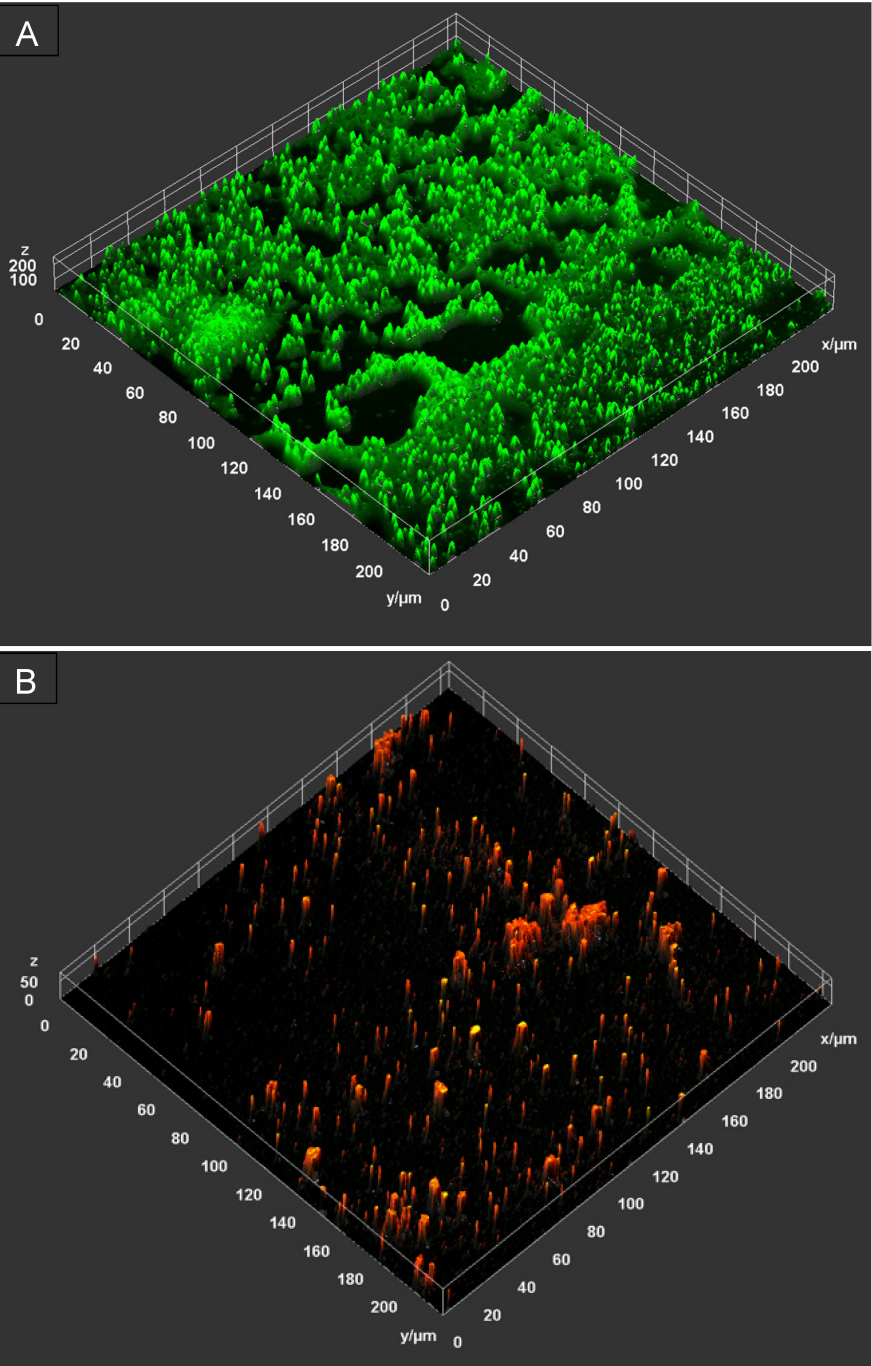
A representative 2.5D microscopy image of the biofilm disruption and inactivation of *P. aeruginosa *biofilm following a 60 minute contact time (n=1). The untreated control (top) demonstrates the abundance of live/viable bacterial cells, as indicated by the green signal, forming microcolonies across the surface, whilst the sample treated with PHMB-poloxamer (bottom) reveal a reduced cell viability (no green signal, little yellow signal as overlay of red and green) and increased abundance on non-viable cells, as indicated by the red signal.

The antibiofilm activity of PHMB-poloxamer irrigation solution was also imaged using *P. aeruginosa* biofilms grown within chamber slides and CLSM. The representative image demonstrates the inactiviation of the bacterial cells as a result of exposure to the solution, as indicated by the noteably lack of viable cells, stained green with Syto-9, in the treated samples in comparison to the untreated samples (Figure 3 (Fig. 3)). There are possible signs of disruption to biofilm formation as evidenced by a reduction in the overall thickness of the biofilm where structural components have been affected.

## Discussion

SSIs are a major contributing factor in patient morbidity and mortality following orthopaedic surgery. As a result, prior to skin closure the wounds are routinely cleansed with irrigation solutions to prevent SSI [20]. An ideal irrigation solution is one with strong antimicrobial activity at low concentration and the ability for biofilm eradication equivalent to 99.9% (lg 3) reduction, whilst exhibiting minimal to no toxic effects on the patient’s wound cells [21], [22], [23], [24]. Irrigation solutions may be used prophylactically to prevent or disrupt early stages of biofilm formation and therefore, must function effectively against a variety of both planktonic and sessile microorganisms [25]. The data in this study demonstrates the inactivation of five clinically relevant opportunistic pathogenic species by commonly used irrigation solutions. Those containing PHMB, in most instances, exhibiting superior antimicrobial activity. In addition, the therapeutic indices determined indicate a much better balance between antimicrobial activity and cytotoxicity for the irrigation solutions containing PHMB (Table 3 (Tab. 3)). In this study, PREVENTIA^®^ Surgical irrigation, Prontosan™ and Lavasorb™ all utilise PHMB as an active ingredient within their irrigating formulations.

PHMB is widely used as an antiseptic for biotic and abiotic surfaces; applications include mouthwash solutions [26], [27], contact lens washes and wound care[28]. Moreover, PHMB has reported efficacy towards both Gram-positive, such as* S. epidermidis* and *Enterococcus faecalis* and Gram-negative bacteria, such as *Escherichia coli* [24], [26], as well as yeasts, such as *Saccharomyces cerevisiae* [29], [30], [31]. For PREVENTIA^®^ Surgical irrigation and Prontosan™, the time kill assay results indicate positive and fast-acting antimicrobial performance upon exposure to the planktonic challenge (approximately 1 min), while Lavasorb™ required a prolonged time to achieve inactivation in comparison (≥30 minutes for complete inactivation). The speed of inactivation is crucial for inhibiting the adhesion of planktonic microbes and thus prevention of biofilm formation. The different materials present in orthopaedic surgery, such as titanium, polyethylene, hydroxyapatite and ceramics, have been shown to become easily colonised by bacteria or yeasts to form biofilms. This can typically occur in three ways: intraoperative inoculation during the initial perioperative period; through haematogenous infection, with microorganisms originating from different parts of the body such as a skin or urinary tract, if infected; and direct transmission from nearby infected tissues [32]. On these surfaces, biofilm forming microorganisms can express an array of phenotypes that alter the biofilms tolerance and susceptibility to antimicrobial agents and therefore it is key to assess the efficacy of irrigation solutions against biofilm communities [33]. The difference between the tested irrigation solutions, PREVENTIA^®^ Surgical irrigation and Prontosan™ on the one hand and Lavasorb™ on the other hand, is the concentration of PHMB present in the solution. While PREVENTIA^®^ Surgical irrigation and Prontosan™ use a concentration of 0.1% PHMB, Lavasorb contains only 0.04% PHMB. Additionally, the clinical study from Müller et al. [14] showed no effect in the reduction of SSI with intraoperative irrigation using an irrigation solution containing 0.04% PHMB compared with saline or no irrigation. It could be hypothesised that the use of a 0.1% PHMB-containing irrigation solution provides a better result in the prevention of SSI, which is supported by the data presented here for time of kill shown in Figure 2 (Fig. 2).

Irrigation solutions are classified as medical devices in Europe. As such, the main effect of the irrigation solution must come from the mechanical side of the action during rinsing [10]. It could be shown that the act of irrigating a wound for the prevention of SSI has an effect independent of the product used [3]. The use of antimicrobials such as PHMB could be considered as an additive effect, helping to prevent infection and biofilm formation, or to help to reduce the load of biofilm that is already established. As seen in the microscopic images, the rinsing solution used, containing 0.1% PHMB, was able to reduce the biofilm within an incubation time of 1 hour. Only a minimum of microcolonies remain after the treatment. In addition, the remaining bacterial cells were found to be largely inactivated, possibly as a result of the active ingredients in the rinse solution, as shown by the viability staining.

We acknowledge, the incubation time of 1 hour used in the experiments does not represent the incubation time in practical use during surgical intervention. However, it does postulate the potential of a combined mode of action using the mechanical effect of irrigation and the antimicrobial effect of the solutions components. Further studies, including those in a clinical context, are needed to improve our knowledge base. PHMB has been shown to be effective against all ESKAPE pathogens, including those tested here with no reported indications of resistance developing [34], [35], [36]. 

The three tested irrigation solutions containing PHMB differed in concentration and in the surfactant they were mixed with PREVENTIA® Surgical irrigation utilises a poloxamer, a non-ionic surfactant. The surfactant is comprised of hydrophobic and hydrophilic groups enabling it to adhere to cellular debris/foreign material and enabling the product to be washed off, respectively. Prontosan™ and Lavasorb™ also incorporate surfactant betaine and macrogolum 400. Surfactants have been shown to inhibit biofilm formation, and effect biofilm detachment [16], [37], [38]. Surfactants allow higher levels of antimicrobial activity at lower concentrations of antiseptic agents [16]. This is important in an environment where antimicrobial and antibiotic stewardship is a significant challenge for healthcare providers; Therefore, in addition to PREVENTIA^®^ Surgical irrigation demonstrating the more favourable average TI value, in this specific study, than Prontosan™ and Lavasorb™ (4.56 vs 3.18 and 3.56), PREVENTIA^®^ Surgical irrigation required lower concentrations (w/w%) to achieve completion inactivation of bacterial growth during MBC assays across all species tested. It is postulated the surfactant may have aided this process.

It is key for irrigation solutions used for wound care to have as little cytotoxic effects as possible on the site being cleansed and not significantly impede processing wound healing [39]. In comparison to other antiseptics, such as povidone iodine, chlorohexidine, and sulfadiazine [40]; often these types of antiseptics are reported to have relatively low therapeutic indices, ranging from 0.5 to 3.0 [41]. PHMB has been reported to have a comparatively superior biocompatibility and reportedly exhibits a potential low-level of cell toxicity during treatment. [42], [43]. As a result, PHMB is considered a relatively safe compound with studies showing limited adverse effect on fibroblasts and keratinocytes in wounds [44]. The combination with the amphoteric surfactant undecylenamidopropyl betaine increases bactericidal efficacy while reducing cytotoxicity by up to 50% [45], improves the cleaning effect [46] and increases the efficacy against biofilms [47]. In this study, PREVENTIA^®^ Surgical irrigation is demonstrated to have the second highest (on average) TI when compared with commonly used irrigation solutions evaluated in this study indicating the solution is more toxic towards the tested pathogens than mammalian cells. The values obtained here are comparable to those produced by Müller and Kramer [40], for PREVENTIA^®^ Surgical irrigation and Betaisonda™. Only Granudacyn™, containing hypochlorous acid, showed a higher therapeutic index in comparison to PREVENTIA^®^ Surgical irrigation. This aligns with literature since the solution used is commonly considered non-cytotoxic but exhibits a low antimicrobial efficacy compared with irrigation solutions containing 0.1% PHMB (Table 1 (Tab. 1)). These findings are consistent with other study results and can be explained by the low concentration of chlorine ions and relatively neutral pH of the irrigation solution used [44]. The results presented by Kamaruzzaman et al. [44] indicate that the microbicidal effects of hypochlorous acid solutions are almost always associated with cytotoxic side effects. For example, depend efficacy and biocompatibility of hypochlorous acid solutions on their physicochemical properties, which also show a strong pH sensitivity to the chlorine concentration present. However, these chloride ions are mainly necessary for its biocidal activity but are also responsible for the cytotoxic effects [48]. In an environment with a high concentration of biological load, no or low antimicrobial effect of hypochlorous acid could be detected against *S. aureus *and* P. aeruginosa* [49]. Wound management is multifaceted and can significantly benefit from products which exhibited one or more therapeutic properties. There are several studies within the literature which indicate PHMB treatment had led to a positive response to the wound healing process [50], [51], with reduced signs of inflammation and accelerated healing [52]. In 2020, Strobel et al. [53] demonstrated in a randomised clinical trial that a PHMB solution was an effective antiseptic in reducing SSI incidents in elective laparotomies. 

## Conclusions

This study highlights the effectiveness of irrigation solutions including PHMB as an active component, against an array of clinically relevant opportunistic pathogenic microbes in the planktonic and sessile, or biofilm state; with the greatest reduction observed for MRSA (5-lg). The mode of action reported for PHMB on bacteria includes disruption of the cell membrane, increased membrane permeability and DNA condensation, whereby the DNA molecules compact affecting further transcription processes [19], [35]. Antibiofilm properties have been readily shown for PHMB solutions, particularly in wound biofilm models [54]. This specific study and data reported here supports the current literature for wound irrigation using PHMB and poloxamer and their use as cleansing solutions during surgical procedures, highlighting PHMB as a highly effective antimicrobial agent.

## Notes

### Competing interests

The authors declare that they have no competing interests.

### Funding

This study was supported by Paul Hartmann AG and as such all the test materials used were supplied to 5D Health Protection Group by Paul Hartmann AG.

## Figures and Tables

**Table 1:**
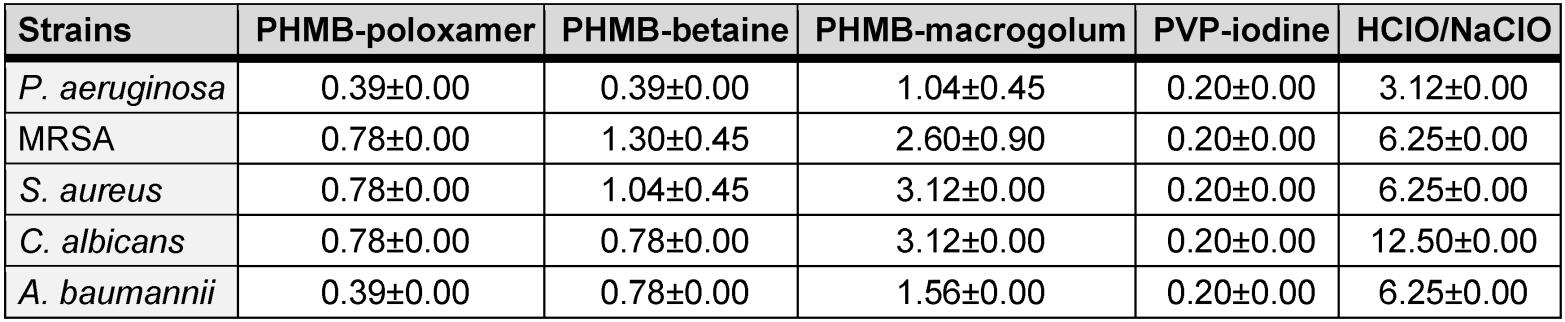
Average minimum bactericidal concentration (%) (n=3) for each test irrigation solution against *P. aeruginosa*, MRSA, *S. aureus, C. albicans*, and *A. baumannii* (the error is reported as the standard deviation of the average)

**Table 2:**
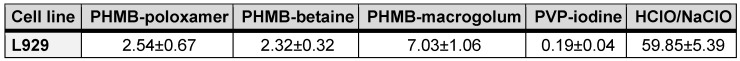
Average concentration (%) (n=3) to achieve 50% cytotoxicity for each test irrigant).

**Table 3:**
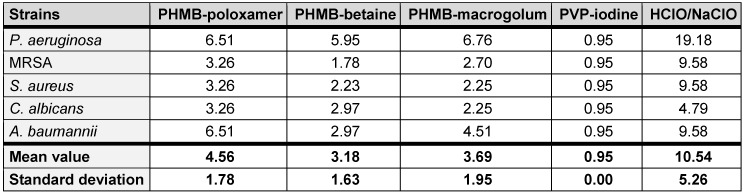
Therapeutic indices (TI) for each test irrigation solution against *P. aeruginosa, *MRSA*, S. aureus, C. albicans, *and* A. baumannii*
